# High Light Induced Alka(e)ne Biodegradation for Lipid and Redox Homeostasis in Cyanobacteria

**DOI:** 10.3389/fmicb.2020.01659

**Published:** 2020-07-17

**Authors:** Yue Qiao, Weihua Wang, Xuefeng Lu

**Affiliations:** ^1^Key Laboratory of Biofuels, Qingdao Institute of Bioenergy and Bioprocess Technology, Chinese Academy of Sciences, Qingdao, China; ^2^Shandong Provincial Key Laboratory of Synthetic Biology, Qingdao Institute of Bioenergy and Bioprocess Technology, Chinese Academy of Sciences, Qingdao, China; ^3^College of Life Science, University of Chinese Academy of Sciences, Beijing, China; ^4^Shandong Provincial Key Laboratory of Energy Genetics, Qingdao Institute of Bioenergy and Bioprocess Technology, Chinese Academy of Sciences, Qingdao, China; ^5^Dalian National Laboratory for Clean Energy, Dalian, China; ^6^Laboratory for Marine Biology and Biotechnology, Qingdao National Laboratory for Marine Science and Technology, Qingdao, China

**Keywords:** alka(e)ne biodegradation, cyanobacteria, high light, lipid, fatty acid, oxidative stress, reactive oxygen species

## Abstract

Cyanobacteria are the oldest photosynthetic microorganisms with good environmental adaptability. They are ubiquitous in light-exposed habitats on Earth. In recent years, cyanobacteria have become an ideal platform for producing biofuels and biochemicals from solar energy and carbon dioxide. Alka(e)nes are the main constituents of gasoline, diesel, and jet fuels. Alka(e)ne biosynthesis pathways are present in all sequenced cyanobacteria. Most cyanobacteria biosynthesize long chain alka(e)nes via acyl-acyl-carrier proteins reductase (AAR) and aldehyde-deformylating oxygenase (ADO). Alka(e)nes can be biodegraded by a variety of cyanobacteria, which lack a β-oxidation pathway. However, the mechanisms of alka(e)ne biodegradation in cyanobacteria remain elusive. In this study, a cyanobacterial alka(e)ne biodegradation pathway was uncovered by *in vitro* enzyme assays. Under high light, alka(e)nes in the membrane can be converted into alcohols and aldehydes by ADO, and aldehyde dehydrogenase (ALDH) can then convert the aldehydes into fatty acids to maintain lipid homeostasis in cyanobacteria. As highly reduced molecules, alka(e)nes could serve as electron donors to further reduce partially reduced reactive oxygen species (ROS) in cyanobacteria under high light. Alka(e)ne biodegradation may serve as an emergency mechanism for responding to the oxidative stress generated by excess light exposure. This study will shed new light on the roles of alka(e)ne metabolism in cyanobacteria. It is important to reduce the content of ROS by optimization of cultivation and genetic engineering for efficient alka(e)ne biosynthesis in cyanobacteria.

## Introduction

Cyanobacteria are the oldest organisms that perform oxygenic photosynthesis similar to what is seen in higher plants. Ancient cyanobacteria played an important role in changing the Earth’s atmosphere from an anoxic to oxic atmosphere. They are ubiquitous in light-exposed habitats on Earth, including extreme environments such as deserts, rocks, salt lakes, hot springs, and polar regions. During their evolution, cyanobacteria have acquired diverse survival abilities that enable them to acclimate to the stress conditions.

Light serves as an energy source for cyanobacteria and plays an essential role in photosynthesis. Cyanobacteria are often exposed to light of different intensities in their natural habitats. High light intensities may destabilize the balance between energy supply and consumption. The excess energy could cause partial reduction of oxygen to generate reactive oxygen species (ROS), such as singlet oxygen (^1^O_2_), hydrogen peroxide (H_2_O_2_), superoxide anion (O${}_{2}^{-}\cdot$) and hydroxyl radical (OH⋅), which would lead to oxidative stress (Latifi et al., 2009; Muramatsu and Hihara, 2012).

All cyanobacteria in light-exposed habitats are frequently subjected to ROS, as they are inevitably generated due to the fluctuations in light intensity. Acclimation mechanisms to ROS include the synthesis of antioxidant enzymes such as superoxide dismutase (Herbert et al., 1992; Inupakutika et al., 2016) catalases (Tichy and Vermaas, 1999; Mironov et al., 2019) and peroxidases (Yamamoto et al., 1999; Mironov et al., 2019) and the enhanced production of antioxidants such as carotenoids (Zhu et al., 2010; Magdaong and Blankenship, 2018), tocopherol (Maeda et al., 2005; Sakuragi et al., 2006; Mene-Saffrane, 2018), and glutathione (Cameron and Pakrasi, 2010) which can scavenge ROS. Since the ROS inevitably damage the membrane lipids of cyanobacteria, there must be some synergetic systems involved in the maintenance of lipid and redox homeostasis in cyanobacteria.

There are three membrane systems, namely, outer membranes, plasma (inner) membranes, and thylakoid membranes, in cyanobacteria. The photosynthesis reactions occur in the thylakoid membranes (Anderson, 1986; Mullineaux, 2014). The plasma and outer membranes facilitate the transport of ions and cofactors across the membranes by porins and permeases (Laudenbach and Grossman, 1991). Therefore, membrane lipids are pivotal for the photosynthesis of cyanobacteria. Lipid peroxidation damage can be caused by ROS, which can react with biologically important molecules such as lipids, DNA, and proteins.

Alka(e)nes are highly reduced and stable molecules. A wide variety of organisms, such as some plants, insects, and microorganisms, can biosynthesize alka(e)nes (Howard and Blomquist, 2005; Schirmer et al., 2010; Bernard and Joubes, 2013). Alka(e)nes in plants can form a cuticle, acting as a protective barrier against biotic and abiotic stresses (Bernard and Joubes, 2013). Insects can synthesize alka(e)nes and secrete them onto the cuticles as a waterproof barrier and as a signaling pheromone. Alka(e)nes in eukaryotic microalgae can act as storage intermediates in the biosynthesis of epoxides and other lipids (Metzger and Casadevall, 1989). The alka(e)nes in cyanobacteria can modulate the cyclic electron flow to facilitate cell growth under cold stress (Berla et al., 2015). Alka(e)nes can accumulate in the cytoplasmic and thylakoid membranes to facilitate curvature and promote membrane flexibility potentially (Lea-Smith et al., 2016).

Alka(e)ne metabolism is ubiquitous in cyanobacteria, regardless of habitat or morphology (Klahn et al., 2014). Schirmer et al. (2010) found that most cyanobacteria biosynthesize long-chain alka(e)nes (C_15_ and C_17_) via acyl-acyl-carrier proteins (ACP) reductase (AAR) and aldehyde-deformylating oxygenase (ADO). A variety of cyanobacteria, such as *Synechocystis* strain UNIGA, *Nostoc punctiforme*, and *Spirulina platensis* (Al-Hasan et al., 1998; Abed and Koster, 2005; El-Sheekh and Hamouda, 2014) are capable of degrading alka(e)nes. However, the alka(e)ne biodegradation pathways in cyanobacteria have not been identified. Previous results have shown that ADO can incorporate an oxygen atom into the alkane to generate alcohol and aldehyde, which indicates that ADO has alkane monooxygenase activity and could catalyze the key reaction of alka(e)ne biodegradation in cyanobacteria (Aukema et al., 2013). In addition, an aldehyde dehydrogenase (ALDH, EC 1.2.1.5) encoded by *slr0091* in *Synechocystis* sp. PCC 6803 (hereafter Syn6803) can convert aldehydes to the corresponding fatty acids. It was shown that the transcript level of *ado* increased notably, while transcript level of *aar* remained unchanged in Syn6803 under high light (Mitschke et al., 2011). The transcript level of *aldh* in Syn6803 also showed significant increase under high light and oxidative stress by real-time RT-PCR and transcriptomic analysis, respectively (Trautmann et al., 2013; Hernandez-Prieto et al., 2016).

The ubiquity of alka(e)ne metabolism has been observed in many photosynthetic organisms; however, the specific roles of alka(e)nes in cyanobacteria remain elusive. In this study, using *in vitro* enzymatic assays, we characterized a cyanobacterial ADO-ALDH alka(e)ne biodegradation pathway. Genetic and physiological analyses showed that alka(e)nes can serve as both fatty acid and electron sinks for maintaining lipid and redox homeostasis in cyanobacteria under oxidative stress. This study sheds new light on the relationship between alka(e)ne biodegradation and the ROS caused by high light.

## Materials and Methods

### Strains and Culture Conditions

*Escherichia coli* (*E. coli*) DH5α and BL21(DE3) grown in Luria Bertani (LB) medium with the corresponding antibiotics under standard concentrations were used for DNA cloning and protein expression, respectively. When required, ampicillin, kanamycin, spectinomycin, and gentamicin were added to final concentrations of 100, 50, 50, and 5 μg/mL, respectively. The genomic DNA of *Synechococcus elongatus* PCC 7942 (hereafter Syn7942) and *Nostoc punctiforme* PCC 73102 (hereafter Nos73102) were extracted and used as the PCR template to clone the corresponding ado and aldh gene. *Synechococcus elongatus* UTEX 2973 (hereafter Syn2973) and a Syn7942 mutant with a single amino acid substitution in its ATP synthase (Sye7942-C252Y) were cultivated under high light (500 μmol photons m^–2^s^–1^) and used to extract the aliphatic compounds. Syn6803 and the mutant strains were cultivated in BG-11 medium (Stanier et al., 1971) at 30°C under continuous illumination by white light (approximately 30 μmol photons m^–2^s^–1^) and bubbled with a stream of air. When required, spectinomycin and gentamicin were added to final concentrations of 50 and 5 μg/mL, respectively. Pre-cultures of Syn6803 and the mutant strains in the exponential phase were diluted to an OD of 0.05 at 730 nm in 300 mL BG-11 medium. Strains were cultivated at 30°C, with bubbling air under normal light (NL: 30 μmol photons m^–2^.s^–1^) or high light (HL: 300 μmol photons m^–2^s^–1^). All strains used in this work are described in Table 1. Details of the plasmids used to construct the mutant strains are included in the Supplementary Experimental Procedures and Supplementary Figures 1, 2. The primers used in this work are described in Supplementary Table 1.

**TABLE 1:** Strains used in this work.

Strains	Genotype	Source or study
QY8	*E. coli* BL21(DE3) with pET28a-*Synpcc7942_0489*	This study
QY9	*E. coli* BL21(DE3) with pET28a-*Npun_F0840*	This study
JZ8	*E. coli* BL21(DE3) with pET28a-*Npun_R1711*	Our laboratory
JZ17	*E. coli* BL21(DE3) with pET28a-*Synpcc7942_1593*	Our laboratory
Syn6803,	*Synechocystis* sp. PCC 6803	Prof. Xudong Xu
Syn7942,	*Synechococcus elongatus* PCC 7942	Prof. Xudong Xu
Syn2973,	*Synechococcus elongatus* UTEX 2973	UTEX^a^
Sye7942-C252Y,	*Synechococcus elongatus* PCC 7942 mutant harboring AtpA with the C252Y point mutation	Our laboratory
Nos73102	*Nostoc punctiforme* PCC 73102	Prof. John C. Meeks
QY4	*Synechocystis* sp. PCC 6803 with *slr0168::Sp-PcpcB-sll0208-sll0209*	This study
QY7	*Synechocystis* sp. PCC 6803 with *slr0168::Sp-PcpcB-sll0208-sll0209 slr1556::Gm-PcpcB-slr0091*	This study

*^*a*^The UTEX Culture Collection of Algae at the University of Texas at Austin.*

### Expression and Purification of the Recombinant Proteins

Aldehyde-deformylating oxygenase (*Synpcc7942_1593*) and ALDH (*Synpcc7942_0489*) from Syn7942, ADO (*Npun_R1711*) and ALDH (*Npun_F0840*) from Nos73102 were overexpressed in *E. coli* BL21(DE3) in LB medium with an N-terminal histidine tag in pET28a (Novagen, Germany). Protein expression was induced using 0.2 mM IPTG at 18°C overnight when the absorption of the cultures at 600 nm reached 0.6–0.8. Notably, 50 μM ferrous ammonium sulfate (Sigma-Aldrich, United States) was required during the expression of ADO to increase the iron content of this protein. Cultures were harvested by centrifugation at 5000 × *g* for 10 min at 4°C and then resuspended in binding buffer (50 mM sodium phosphate, 0.3 M NaCl, pH 7.2). Cells were disrupted by sonication in an ice water mixture. Binding buffers containing various concentrations of imidazole (5–20 mM) were used to wash the recombinant proteins, and 250 mM imidazole was used to elute the recombinant protein at 4°C. The purified protein was desalted by dialysis against 50 mM sodium phosphate, pH 7.2. A 12% polyacrylamide gel was used for SDS-PAGE, and Coomassie blue R-250 was used for staining. The proteins were concentrated, and the concentration of protein was determined by the Bradford method using bovine serum albumin as the standard.

### Enzyme Assays and Quantitation of Products by GC-MS

The ADO and ALDH enzyme assays were carried out in 500 μL reactions (Lo and Chen, 2010; Aukema et al., 2013). The reaction mixture contained 50 mM potassium phosphate buffer (pH 7.2), 80 μM ferrous ammonium sulfate, 75 μM phenazine methosulfate (PMS) and 2 mM NADH along with 500 μM aldehyde substrate. The reaction was initiated by adding 30 μM ADO, maintained at 25°C for 30 min at 100 rpm, and terminated by transferring the reaction vials into an ice bath. To initiate the other reaction, 1 mM dithiothreitol (DTT), 2 mM NAD^+^ and 1 μM ALDH enzyme were added together at 25°C, and the reaction was continued for another 30 min at 100 rpm. The reaction mixtures were extracted with 500 μL of methyl tert-butyl ether (MTBE). The organic layer was analyzed by gas chromatography–mass spectrometry (GC-MS) using an Agilent 7890A-5975C system equipped with an HP-5 ms column (100% dimethylsiloxane capillary; 30 m × 250 μm × 0.25 μm). Helium was used as the carrier gas with a flow rate of 1 mL/min, and the injection port was maintained at 250°C. The following oven temperature program was applied: 40°C held for 1 min, ramped to 200°C at 5°C/min, then increased to 240°C at 25°C/min, and finally held for 15 min.

### Detection of ROS Production *in vivo* Using DCFH-DA

A 2′,7′-Dichlorodihydrofluorescein diacetate (DCFH-DA) (Solarbio, China) is an oxidant-sensing fluorescent probe used for detecting intracellular ROS. DCFH-DA, which shows no fluorescence, can be converted into DCFH by intracellular esterase, and then DCFH can be oxidized by intracellular ROS to 2′,7′-dichlorofluorescein (DCF), which is highly fluorescent at an excitation wavelength of 488 nm and an emission wavelength of 525 nm. Thus, the level of intracellular ROS can be quantified by detecting the fluorescence intensity of the DCF. To detect the intracellular ROS level, a 10 mM (w/v) stock solution of DCFH-DA in ethanol was prepared and kept at −20°C in a brown, airtight vial until use. The DCFH-DA stock solution was diluted with PBS at a ratio of 1:1000 to a final concentration of 10 μmol/L. Cells at 1 OD_730_ under high light at different time points were collected and suspended in 1 mL of 10 μmol/L DCFH-DA and then incubated for 20 min in a 37°C shaker to ensure that the cells are fully exposed to the probe. Cells were washed three times with PBS to sufficiently remove any DCFH-DA that did not enter the cells. Finally, the cells were resuspended in 200 μL of PBS. Then, the fluorescence of DCF was detected with a microplate reader.

### Photochemical Efficiency Measurements (Fv/Fm)

The chlorophyll fluorescence parameter was measured by pulse amplitude modulation (PAM) fluorometry (Dual-PAM-100, Heinz Walz, Germany). The sample volume was 2 mL, and after treatment in the dark for 5 min, and Fo (minimum fluorescence) and Fm (maximum fluorescence) were measured. The maximum quantum yield of photosystem II (Fv/Fm) was calculated based on the following formula: Fv/Fm = (Fm-Fo)/Fm.

### Peroxidation of Membrane Lipids Under High Light

Malondialdehyde (MDA) can be used as an indicator of lipid peroxidation in biological systems under high light conditions (Mihara and Uchiyama, 1978; Jantaro et al., 2006). The MDA level in this study was measured using a lipid peroxidation assay kit (Sigma-Aldrich, United States). Cells at OD_730_ = 1 under high light were collected, and 300 μL of MDA lysis buffer was added to homogenize the samples. The homogenate was centrifuged at 13,000 × *g* for 10 min to remove insoluble material, and the membrane lipid peroxidation level was measured in the supernatant. The MDA–thiobarbituric acid (TBA) adduct was formed by adding 600 μL of the TBA solution to each vial containing sample and incubating the samples at 95°C for 60 min. The samples were cooled to room temperature in an ice bath for 10 min, and then a 200 μL aliquot of each reaction mixture was transferred to a 96-well plate for analysis. The MDA–TBA adduct fluoresces with an excitation wavelength of 532 nm and an emission wavelength of 553 nm. Thus, the peroxidation of the membrane lipids was measured based on a standard curve of MDA.

### Alka(e)ne Extraction and GC-MS Analysis

Cultures (80 mL) were harvested at 8000 rpm for 10 min, resuspended in 15 mL of ultra-pure water and then lysed by sonication. Nonadecanoic acid (50 μL, 1.5 mg/mL) and 50 μL of eicosane (1 mg/mL) were added to the cell lysis solution as internal standards for alka(e)ne analysis. Chloroform–methanol (10 mL, 2:1, v/v) was added to the lysate, and the mixture was extracted for 2 h at room temperature. After shaking for 2 h, a two-phase system (top: aqueous, bottom: organic) was generated by centrifugation at 8000 rpm at room temperature for 15 min. The organic phase was transferred to a new clear glass tube and then concentrated to dryness under a stream of nitrogen at 55°C. The residue was dissolved in 1 mL of n-hexane and analyzed using an Agilent 7890A-5977C GC-MS system equipped with an HP-INNOWax column (30 m × 250 μm × 0.25 μm). Helium was used as the carrier gas at a flow rate of 1 mL/min, and the temperature of the injection port was 250°C. The following temperature program was applied to analyze the extract: 40°C for 1 min, increase at 5°C/min to 200°C, then increase at 25°C/min to 240°C, and finally hold for 15 min. The internal standard was used to determine the yield of alka(e)ne and free fatty acid, which were reported as the mean from three independent experiments.

### Total Lipid Extraction and GC-MS Analysis

Sufficient cell cultures were harvested by centrifugation at 10,000 rpm for 5 min, and the pellets were lyophilized for 40 h. Cyanobacteria powders (20 mg) were transferred to 15 mL centrifuge tubes. BG-11 medium (2 mL) and acetic acid (200 μL) were added to the powders, and the tubes were shaken to mix thoroughly. After shaking for 15 min, 50 μL of nonadecanoic acid (1.5 mg/mL) was added to the mixture as the internal standard for fatty acid analysis. The culture was extracted for 1 h at room temperature with 4 mL of chloroform:methanol (1:1, v/v) (Bligh and Dyer, 1959). A two-phase system (top: aqueous, bottom: organic) was generated after shaking for 1 h and centrifuged for 20 min at 8000 rpm at room temperature. The bottom organic phase was transferred to an esterification tube and concentrated to dryness under a stream of nitrogen at 55°C. Then, 2 mL of solution F1 (0.4 M KOH – CH_3_OH) was added to the samples, and these mixtures were then dried under nitrogen in a 60°C water bath for 1 h. After cooling to room temperature, 4 mL of F2 solution (HCl:CH_3_OH = 1:9, v/v) was added, and this mixture was maintained in a 60°C water bath for 20 min. After cooling, 1–2 mL of n-hexane and 3 mL of saturated NaCl solution were added to the esterification tube and mixed as gently as possible for a few minutes. After separation of the two phases, the top organic phase was transferred to an airtight bottle. The samples were used for GC-MS analysis to confirm the kinds and quantities of different fatty acids. The methyl esterified fatty acid samples were assayed with GC-MS using an Agilent 7890A-5977C system equipped with an HP-INNOWAX column (30 m × 250 μm × 0.25 μm). Helium gas was used at a flow rate of 1 mL/min, and the temperature of the injection port was 250°C. The following temperature program was applied: 100°C for 1 min, increase at 5°C/min to 200°C, increase at 25°C/min to 240°C, and finally hold for 15 min. An internal standard was used to determine the yields of the different fatty acids, and the yields are reported as the mean from three independent experiments.

Additional details on methods are provided in Supplementary Experimental Procedures.

## Results

### *In vitro* Enzymatic Assays Uncover a Cyanobacterial ADO-ALDH Alka(e)ne Biodegradation Pathway

Alka(e)nes can be degraded by a wide variety of cyanobacteria, while the alka(e)ne biodegradation pathway has not been identified previously. Interestingly, it has been shown that ADO, the key enzyme in alka(e)ne biosynthesis, is capable of catalyzing the incorporation of an oxygen atom into the alka(e)nes to yield the corresponding alcohol and aldehyde. The ALDH in cyanobacteria can mediate the oxidation of aldehydes into the corresponding fatty acids (Trautmann et al., 2013). In most aerobic alkane biodegradation pathways, alka(e)nes are oxidized to the corresponding primary alcohols by a hydroxylase. The primary alcohol is subsequently further oxidized to the corresponding aldehyde and fatty acid (Wentzel et al., 2007). It was reported that alka(e)nes could be degraded by *Nostoc punctiforme* (El-Sheekh and Hamouda, 2014). The ADO and ALDH from Nos73102 are supposed to be involved in the alka(e)ne biodegradation. The ALDH of Syn6803 and Syn7942 both belong to the class 3 ALDH, which can oxidize aldehydes to the corresponding fatty acids (Kaiser et al., 2013; Trautmann et al., 2013). Heterologous expression of Syn6803 ALDH in *E. coli* results in the formation of insoluble and inactive inclusion bodies. So, we chose ADO and ALDH from Syn7942 and Nos73102 for *in vitro* enzymatic assays (Supplementary Figure 3). Considering the high boiling point of long-chain fatty alcohols (>C_14_) complicates gas chromatography analysis, we first tested substrates with different carbon chain lengths (C_9_, C_12_, and C_14_) and further confirmed that ADO can indeed catalyze the hydroxylation of alka(e)nes (Supplementary Figure 4). These results indicate that ADO may play a pivotal role in alka(e)ne biodegradation.

**FIGURE 1:**
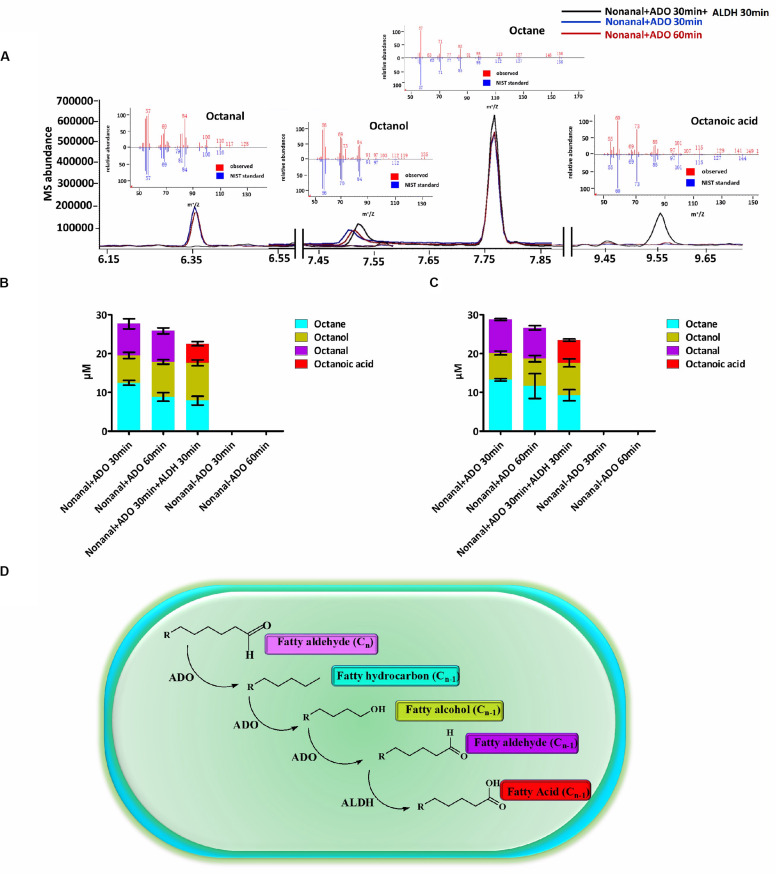
A cyanobacterial ADO-ALDH alka(e)ne biodegradation pathway was identified by *in vitro* enzymatic assays. **(A)** GC-MS chromatograms of Syn7942 ADO-ALDH reaction with nonanal as the substrate. The inset shows the mass spectra of octane, octanal, octanol, and octanoic acid from GC-MS analysis (red) compared with mass spectra from the National Institute of Standards and Technology (NIST) database (blue). **(B)** Product profiles of the Syn7942 ADO-ALDH reaction with nonanal as the substrate. **(C)** Product profiles of the Nos73102 ADO-ALDH reaction with nonanal as the substrate. **(D)** Proposed mechanism for the cyanobacterial ADO-ALDH alka(e)ne degradation pathway. Error bars indicate the standard deviation of triplicate analyses.

Subsequently, we identified an *in vitro* enzyme system that could catalyze the conversion of an aldehyde (C_n_) into an alka(e)ne (C_n–1_), and then to an aldehyde (C_n–1_) by ADO. The aldehyde (C_n–1_) could then be converted to the corresponding fatty acid (C_n–1_) by ALDH. ADO reaction mixtures containing nonanal and a chemical reducing system including NADH and the electron acceptor PMS were extracted with MTBE and analyzed by GC-MS. The octane, octanol, and octanal contents in the mixtures were determined. Then, ALDH was added to the ADO reaction mixtures, and the reactions were incubated for an additional 30 min before extraction. The octanal was consumed and was almost completely converted to octanoic acid by ALDH (Figures 1A–C). The *in vitro* enzyme assays revealed a cyanobacterial ADO-ALDH alka(e)ne biodegradation pathway. ALDH could convert the aldehyde intermediates from the ADO reaction into the corresponding fatty acids (Figure 1D).

### Aldehydes With Severe Cytotoxicity Can Be Detected in High Light-Tolerant Cyanobacteria

Alka(e)ne metabolism is widespread in cyanobacteria (Klahn et al., 2014). It is reported that cyanobacteria are much more tolerant to alka(e)nes than free fatty acids, alcohols, and aldehydes (Kamarainen et al., 2012). In this study, we detected the effects of aliphatic compounds containing 12 carbons (dodecane, dodecanal, dodecanol, and dodecanoic acid) on the growth of Syn6803 in two systems (Supplementary Table 2). We found that dodecane causes minimal damage to cyanobacterial cells even though it was 6.7 times more abundant than dodecanal, dodecanol, and dodecanoic acid. Moreover, after incubation with high concentrations of aliphatic compounds for 5 h, dodecanal caused obvious cell bleaching in Syn6803 even when the amount of aldehyde added was approximately half that of dodecane, dodecanol, and dodecanoic acid (Supplementary Figure 5).

**FIGURE 2:**
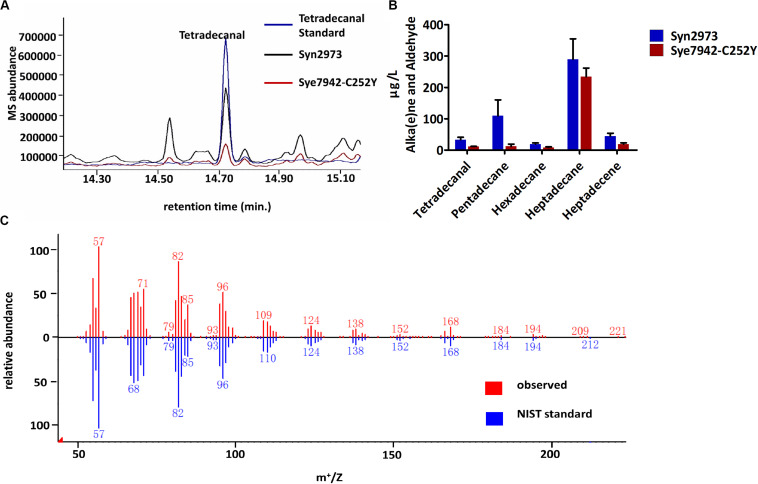
Accumulation of fatty aldehydes in Syn2973 and Sye7942-C252Y under high light. **(A)** Overlaid GC-MS chromatograms of Syn2973 (black line) and Sye7942-C252Y (red line) extracts from cultures under high light with tetradecanal as a standard (blue line). **(B)** Quantification of fatty aldehydes and alka(e)nes in Syn2973 and Sye7942-C252Y cultures grown under high light. **(C)** Mass spectra of tetradecanal from GC-MS analysis (red) of Syn2973 cultures under high light compared with mass spectra from the National Institute of Standards and Technology (NIST) database (blue). Error bars indicate the standard deviation of triplicate analysis.

Aldehyde accumulation is limited by its toxicity and high reactivity in most organisms. It is difficult to obtain enough biomass for aldehyde determination due to the aldehyde toxicity to Syn6803 and Syn7942 strains under high light exposure. In this study, tetradecanal was detected in two high light-tolerant cyanobacterial strains (Figure 2): Syn2973 (Yu et al., 2015) and the Syn7942 mutant Sye7942-C252Y (Lou et al., 2018). Both Syn2973 and Sye7942-C252Y could accumulate substantial biomass under high light. Notably, the genomic sequences of Syn2973 and Syn7942 are 99.8% identical. The ADO, AAR, and ALDH sequences in Syn2973 are the same as those in Syn7942. Resequencing of the Sye7942-C252Y genome ensured that the C252Y mutation in its ATP synthase was the only mutation relative to the Syn7942 genome (Lou et al., 2018).

Moreover, Syn2973 exhibits a significantly reduced phycobilisome content under high light (Ungerer et al., 2018). This also suggested that the aldehydes might cause cell bleaching even in cyanobacterial strains tolerant of high light. Under high light, the ROS level in the cyanobacterial cells may increase due to the partial reduction of oxygen by the excess electrons.

### ROS-Mediated Alka(e)ne Biodegradation for Lipid and Redox Homeostasis in Cyanobacteria

**FIGURE 3:**
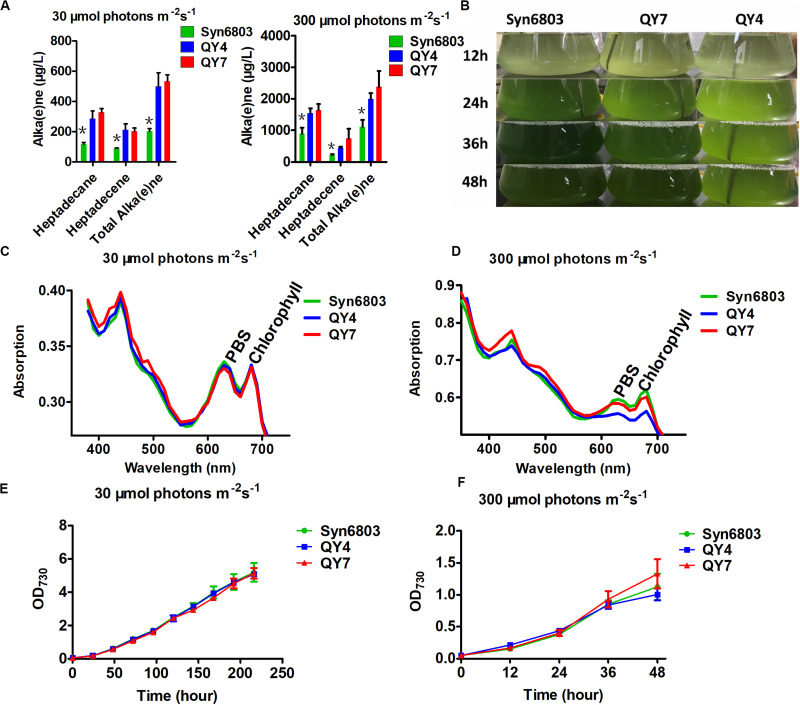
Alka(e)ne and phenotype of cyanobacterial strains under normal and high light. **(A)** Alka(e)ne contents in the three strains under normal and high light conditions. **(B)** Phenotypes of Syn6803, QY4, and QY7 strains under high light as a function of time. **(C)** Whole cell absorption spectra from 350 to 750 nm for Syn6803, QY4, and QY7 strains under normal light for 48 h at 30°C. The absorption spectra were normalized to OD_730_. PBS, phycobilisomes. **(D)** Whole cell absorption spectra from 350 to 750 nm for Syn6803, QY4, and QY7 strains under high light for 48 h at 30°C. The absorption spectra were normalized to OD_730_. PBS, phycobilisomes. **(E)** Growth curve of the three strains under normal light for 9 days. **(F)** The growth curve of the three strains under high light for 48 h. Error bars indicate the standard deviation of triplicate analyses. Asterisks indicate significant differences (Student’s *t* test, *P* < 0.05) of alka(e)ne contents between Syn6803 and the other two strains (QY4 and QY7). There was no significant difference between the alka(e)ne contents of QY4 and QY7 strain.

Two *Synechocystis* mutants related to alka(e)ne metabolism were constructed to verify the specific roles of alka(e)ne biodegradation in cyanobacteria. Alka(e)ne content was not enhanced by only overexpressing AAR or ADO in cyanobacteria (Wang et al., 2013), so one additional copy of both *sll0208* (*ado*) and *sll0209* (*aar*) driven by a strong constitutive promoter *PcpcB* were integrated into the *slr0168* locus, which is normally used as a neutral site in Syn6803 (Williams, 1988) to generate the QY4 strain. On that basis, one additional copy of *slr0091* (*aldh*) was integrated into the *slr1556* site (a redundant D-lactate dehydrogenase homolog) (Angermayr et al., 2016) of the QY4 strain to generate the QY7 strain (Supplementary Figure 2). The Syn6803, QY4, and QY7 strains were cultivated under normal light (30 μmol photons m^–2^ s^–1^) and high light conditions (300 μmol photons m^–2^ s^–1^). The alka(e)ne contents in the QY4 and QY7 strains with two copies of *aar* and *ado* were similar to each other and markedly higher than that of Syn6803 under both normal and high light conditions (Figure 3A).

Under high light, QY4 cells were more strongly bleached than those of the wild-type strain. A time course of *aar* and *ado* overexpression led to the stepwise bleaching of the QY4 strain after 24 h. In the QY7 strain, the overexpression of the additional copy of *aldh* relative to the QY4 strain recovered the bleaching phenotype (Figure 3B). There was no significant difference in photosynthetic pigment contents in Syn6803, QY4, and QY7 strain under normal light conditions at 48 h (Figure 3C). However, the whole cell absorption spectra after 48 h of exposure to high light showed that the amounts of photosynthetic pigments in the QY4 strain were considerably lower (Figure 3D). Similar growth curves of three strains were observed under normal light exposure (Figure 3E). While the growth of the QY4 strain was not as good as that of the Syn6803 and QY7 strains from 24 to 48 h of high light exposure (Figure 3F). These results indicated that the excess alka(e)nes in the QY4 strain could be converted to aldehydes by overexpressed ADO, leading to aggravated cell damage under high light. In the QY7 strain, the accumulated aldehydes can be converted to fatty acids for lipid turnover by overexpressed ALDH, so the cells could recover from high light.

**FIGURE 4:**
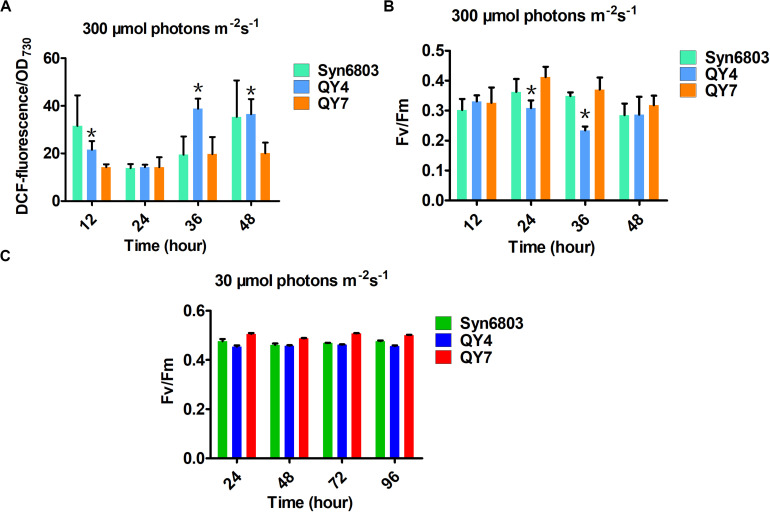
Effects of light intensity on the tested cyanobacterial strains at different time points. **(A)** The intracellular ROS levels in the three tested strains under high light. **(B)** The ratio of Fv/Fm in the three tested strains under high light. **(C)** The ratio of Fv/Fm in the three tested strains under normal light. Error bars indicate the standard deviation from triplicate analyses. Asterisks indicate significant differences (Student’s *t* test, *P* < 0.05) between the data of QY4 and QY7 strain. There was no significant difference between the data of Syn6803 and QY7 strain.

Reactive oxygen species production in WT, QY4, and QY7 strains under high light was examined using DCFH-DA. DCFH-DA is an applicable marker for measuring ROS levels in cyanobacterial cells (Rastogi et al., 2010). Intracellular ROS levels in the QY4 strain were much higher than those in the Syn6803 and QY7 strain after 36 h of high light exposure (Figure 4A). These results indicated that aldehyde accumulation could lead to an increase in the ROS levels, which is thought to aggravate the deleterious effects of aldehydes.

As the ROS level continued to increase, the MDA content, which indicates the degree of lipid peroxidation, also increased with some delay (Table 2). The MDA level in the QY4 strain was relatively higher than that of the Syn6803 after 36 and 48 h of high light exposure. While the MDA level in the QY7 strain was close to that of Syn6803. In addition, we measured the ratio of the variable fluorescence (Fv) to the maximum fluorescence (Fm), which is indicative of the photosynthetic efficiency of the cyanobacterial strain. As the high light treatment was extended beyond 24 h, the Fv/Fm ratio of the QY4 strain decreased. High light treatment for 36 h resulted in a significant decrease in the Fv/Fm ratio for the QY4 strain (Figure 4B). The Fv/Fm ratios of the three strains remained at approximately 0.5 during cultivation under normal light (Figure 4C).

**TABLE 2:** MDA content in the three tested strains under high light.

Time	Strain		
	
	Syn6803^a^	QY4^a^	QY7^a^
12 h	3.670.79	3.880.37	3.290.53
24 h	2.040.1	2.100.28	2.030.2
36 h	3.291.13	4.451	2.940.83
48 h	6.750.43	7.890.7	6.930.41

*Concentrations of MDA are presented in μM/OD_730_. ^*a*^Data are shown as means ± standard deviation (*n* = 3).*

## Discussion

### ADO Plays a Pivotal Role in Alka(e)ne Biodegradation in Cyanobacteria

Aldehyde-deformylating oxygenase is a versatile catalyst in alka(e)ne metabolism in cyanobacteria. ADO, the key enzyme in alka(e)ne biosynthesis, can also catalyze the key reaction in alka(e)ne biodegradation. The enzymes involved in alka(e)ne biosynthesis and biodegradation are generally different (Wang and Shao, 2013; Herman and Zhang, 2016). Cyanobacteria, which are evolutionarily ancient organisms, have relatively small genomes and few encoding genes. Therefore, cyanobacterial enzymes may play versatile physiological and metabolic roles.

The *ado*–*aar* locus and the adjacent region consisting in Syn6803 show an extremely complex organization. Unexpectedly, *ado* and *aar* do not constitute an operon but are expressed independently and follow different regulatory patterns (Klahn et al., 2014). The expression data suggest that ADO may play an additional role in a different functional context (Mitschke et al., 2011).

Aldehyde-deformylating oxygenase belongs to the nonheme diiron family of oxygenases exemplified by soluble methane monooxygenase (sMMO). The crystal structure of ADO is very similar to that of the α subunit of the MMO hydroxylase component (MMOH). The active sites of both ADO and MMO are housed within an antiparallel 4-helix bundle in which the two iron atoms are each coordinated to a histidine and two carboxylate ligands (Marsh and Waugh, 2013). Notably, MMOH can oxidize a wide range of substrates, including C_1_–C_8_ alka(e)nes (Colby et al., 1977; Green and Dalton, 1989).

Methane monooxygenase is much more complicated than ADO. sMMO consists of three components, a hydroxylase (MMOH), which contains the active site, a reductase (MMOR), and a regulatory protein (MMOB) (Lee et al., 2013). MMOH consists of three subunits (α, β, and γ) that form an α2β2γ2 homodimer and the nonheme diiron active site that catalyzes the hydroxylation of methane and other substrates. MMOR plays key roles in transferring reducing power to the active site of MMOH. MMOB is known to regulate the activity of MMOH (Gassner and Lippard, 1999; Walters et al., 1999). The diiron center in ADO cycles between different iron valence states. The presence of ROS is conducive to the generation of high-valent metal ions (Wang et al., 2016). ADO can form a high-valent Fe^IV^Fe^IV^ intermediate similar to compound Q of the MMOH under oxidative stress. The Fe^IV^Fe^IV^ species could activate oxygen and promote the hydroxylation of the initially formed alka(e)ne by a radical mechanism (Aukema et al., 2013). The radicals are formed by the interaction of organic molecules with metal ions. Radical reactions in biological systems take place at the active sites of enzymes containing metal ions (Rajakovich et al., 2015).

Aldehyde-deformylating oxygenase also requires oxygen and reducing power for activity, but proteins analogous to MMOR and MMOB have not yet been identified in cyanobacteria. Hydrogen peroxide (H_2_O_2_) can activate sMMO to catalyze the oxidation of methane and other substrates. MMOR, MMOB, O_2_, and NADH are generally required for the catalysis but can be substituted by H_2_O_2_, which can act as both the O_2_ and electron sources for the reaction (Jiang et al., 1993). H_2_O_2_ is produced as an intermediate in O_2_ reduction by cyanobacteria under oxidative stress (Telor et al., 1986).

### Alka(e)ne Metabolism Is a Versatile System for Modulating Lipid and Redox Homeostasis in Cyanobacteria

As photosynthetic organisms, cyanobacteria are often exposed to fluctuant light intensities in their natural habitat. From night to noon, the outdoor light intensity can range from near 0 to over 2000 μmol photons m^–2^s^–1^. In cyanobacteria, the generation of ROS byproducts by light-driven photosynthetic electron transport is inevitable, especially when the rate of electron transport exceeds that of electron consumption. ROS can cause severe oxidative damage to cellular components. Cyanobacteria have evolved various acclimatory mechanisms to maintain the balance between electron transport and consumption and to protect cells under stress conditions (Latifi et al., 2009). Antioxidant systems in cyanobacteria can effectively cope with enhanced ROS caused by high light. Excess alka(e)nes would not cause significant cyanobacterial cell damage, while accumulated aldehydes may increase cytotoxicity and result in cell bleaching.

Alka(e)nes localize to the cyanobacterial membranes, predominantly to thylakoid membranes (Lea-Smith et al., 2016). Thylakoids are membrane-bound structures embedded in the cytoplasm of cyanobacteria (Liberton et al., 2013). Alka(e)nes localized in the thylakoid membrane may serve as an emergency system to scavenge ROS and boost lipid turnover *in situ*. Several bacterial strains can degrade membrane-bound alkanes by soluble cytochrome P450 monooxygenases (Maier et al., 2001; Van Beilen et al., 2006).

In cyanobacteria, H_2_O_2_ is inevitably generated and then reduced to hydroxyl radicals via the Fenton reaction under oxidative stress. The production of hydroxyl radicals is enhanced in the presence of transition metals. Hydroxyl radicals are highly reactive, and are thus very dangerous to the organism (Sies, 1993). Unlike superoxide, which can be dismutated to O_2_ or H_2_O_2_ by superoxide dismutase, the hydroxyl radical cannot be scavenged by enzymatic catalysis (Reiter et al., 1995).

Alka(e)nes are highly reduced molecules, and excess electrons can be stored as alka(e)nes. The alka(e)ne-deficient *Synechocystis* mutant exhibited enhanced cyclic electron flow (Berla et al., 2015), which indicates that alka(e)nes could serve as electron sinks to decrease electron leak to O_2_. Under oxidative stress, alka(e)nes can act as electron donors to reduce partially reduced ROS. In addition, alka(e)nes can be oxidized to fatty acids. Intracellular alka(e)nes remain at low but constant concentrations in cyanobacteria, which implies ongoing alka(e)ne biosynthesis and biodegradation to maintain homeostasis throughout periodic changes in light intensity.

Alka(e)nes are chemically rather inert and must be activated before they can be metabolized. In most aerobic alka(e)ne degradation pathways, the alka(e)ne is oxidized to the corresponding primary alcohol by alka(e)ne monooxygenases/hydroxylases. The primary alcohol is further oxidized to the corresponding aldehyde and fatty acid. The fatty acid can be conjugated to coenzyme A (CoA) to form a fatty acyl-CoA, which is further processed by β-oxidation to generate acetyl-CoA. It has been proven that cyanobacteria lack a β-oxidation pathway (Taylor, 2012). As photoautotrophic organisms, cyanobacteria do not need to use alka(e)nes as sources of carbon and energy.

**FIGURE 5:**
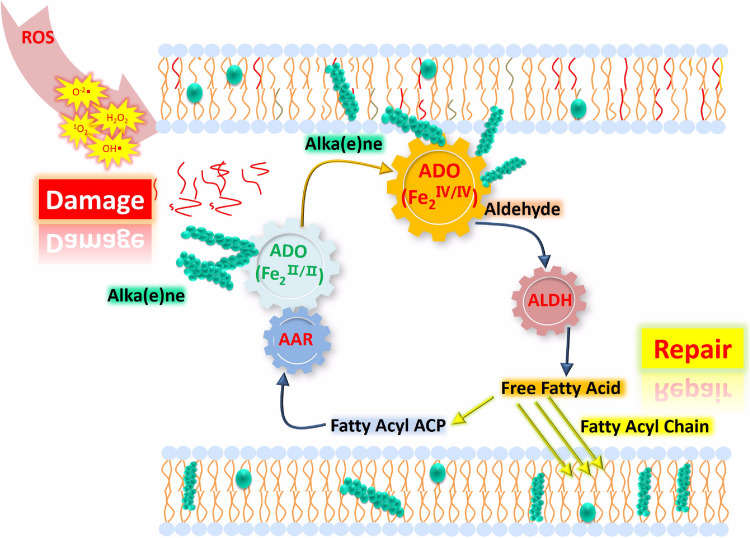
Proposed model of lipid homeostasis via alka(e)ne biodegradation in cyanobacteria under high light. ROS accumulate significantly under high light, which leads to membrane lipid damage (represented as the red lines close to “Damage”) and changes in the iron valence in the ADO active center to form the Fe^IV^ superoxo species. Then, the ADO with Fe^IV^ superoxo species will convert the alka(e)nes (represented as green sphere) to the corresponding aldehydes, which will be catalytically converted to fatty acids by ALDH. The resulting fatty acids will support membrane lipid homeostasis under oxidative stress. Highly reduced alka(e)nes can serve as electron sinks for maintaining redox homeostasis in cyanobacteria. Under high light, alka(e)nes can act as electron donors to reduce the partially reduced ROS.

The correlation between oxidative stress and alka(e)ne degradation provides insight into the physiological roles of alka(e)ne metabolism in cyanobacteria. Alka(e)nes serve as a fatty acid and electron sink for maintaining lipid and redox homeostasis in cyanobacteria. Under oxidative stress, alka(e)nes can be converted to fatty acids by ADO and ALDH to regenerate the acyl chains of damaged lipids and scavenge ROS to maintain redox homeostasis in cyanobacteria (Figure 5).

In cyanobacteria, the transfer of excess electrons to oxygen can generate partially reduced ROS and lead to photodamage of cellular components under oxidative stress caused by high light. Alka(e)nes in cyanobacteria can be oxidized to aldehydes by ADO under high light. Subsequently, the highly reactive aldehyde intermediates can be converted to fatty acids. The fatty acids originating from alka(e)ne biodegradation can then be used for lipid turnover under oxidative stress. As electron sinks, alka(e)nes could simultaneously provide electrons to reduce ROS. Oxidative stress was verified as a critical obstacle to alka(e)ne biosynthesis in cyanobacteria. It is beneficial for the alka(e)ne biosynthesis in cyanobacteria to avoid high light irradiation. Improving the capacity of cyanobacteria to scavenge ROS by genetic engineering of the antioxidant systems will also contribute to alka(e)ne biosynthesis. This study will guide approaches and strategies for efficient alka(e)ne biosynthesis in cyanobacteria.

## Data Availability Statement

All datasets generated for this study are included in the article/Supplementary Material.

## Author Contributions

WW and XL designed the study and interpreted the data. YQ and WW performed the research and analyzed the data. All authors drafted and edited the manuscript, read and approved the final manuscript.

## Conflict of Interest

The authors declare that the research was conducted in the absence of any commercial or financial relationships that could be construed as a potential conflict of interest.
