# Supplementary material for: High Light Induced Alka(e)ne Biodegradation for Lipid and Redox Homeostasis in Cyanobacteria
Source: Front Microbiol. 2020 Jul 17;11:1659. doi: 10.3389/fmicb.2020.01659 (PMC7379126; doi:10.3389/fmicb.2020.01659)
Supplement: Supplementary file 1 [file Data_Sheet_1.docx]

**Supplementary Material**

**Supplementary Experimental Procedures**

**Plasmid and Mutant Construction**

The genomic DNA of Syn6803, Syn7942 and Nos73102 were used as the template for PCR. All primers used were listed in the **Supplementary Table 1**. For ALDH expression vector construction in E. coli BL21(DE3), the *Synpcc7942_0489* from Syn7942 was amplified using gene specific primers (**Supplementary Table 1**) and inserted into the *pEASY*-Blunt Simple Cloning Vector (Transgen, Beijing, China), then subcloned into the pET28a which was digested with *Sac*Ⅰ and *Hind*Ⅲ to form pQY2*.* Similarly, the *Npun_F0840* was subcloned into the pET28a digested with *Sac*Ⅰ and *Sal*Ⅰ to form pQY3. The plasmid of pQY2 and pQY3 was transformed into E. coli BL21(DE3) to generate ALDH expression strain QY8 and QY9, respectively (Table 1).

To construct Synechocystis mutant strains, the endogenous promoter PcpcB of Syn6803 phycocyanin beta subunit was amplified using the genomic DNA of Syn6803 with gene specific primers (**Supplementary Table 1**) and assembled in *pEASY*-Blunt Simple Cloning Vector to form pQY4 plasmid. Then, pQY4 and pLX3 (**Supplementary Figure 1A**) were digested with *Sal*Ⅰ and *Xba*Ⅰ, respectively. The promoter region was subcloned into pLX3 vector to form pQY1 plasmid (**Supplementary Figure 1B**) which was used to transform Syn6803 to construct QY4 mutant strain.

To construct the ALDH overexpression mutant in Syn6803, aldh (slr0091) was amplified using gene specific primers (**Supplementary Table 1**) and similarly the gene fragment was cloned into *pEASY*-Blunt Simple Cloning Vector to form pQY6 plasmid. Then pQY6 and pZZ076 (**Supplementary Figure 1C**) were digested by *Nde*Ⅰ and *Spe*Ⅰ, respectively. The *slr0091* fragment was subcloned into the digested product of pZZ076 to form pQY7 plasmid (**Supplementary Figure 1D**). Then the pQY7 plasmid was digested with *Xba*Ⅰ and *Spe*Ⅰ, and the pZZ089 plasmid (**Supplementary Figure 1E**) was digested only with *Spe*Ⅰ*.* Finally, the fragment of Gentamicin (Gm)-PcpcB-Slr0091 from pQY7 was inserted into the pZZ089 plasmid digested with *Spe*Ⅰ to generate pQY5 plasmid (**Supplementary Figure 1F**) which was used to transform the QY4 to construct QY7 strain. In Supplementary Table 1, the primer 0168-1/0168-2 and ddh-F/ddh-R were used as checking primers for pQY1 and pQY5 construction, respectively.

**Enzyme Assays of ADO Reactions**

For further confirmation the hydroxylase activity of ADO, according to the published procedure , enzyme assays were carried out in 500 μL reaction volumes in 1.5 mL GC vials shaken at 100 rpm at room temperature for 30 min. Reactants included 100 mM HEPES (pH7.4) , 80 μM ferrous ammonium sulfate, chemical electron transfer system 75 mM phenazine methosulfate (PMS) with 2 mM NADH, 500 μM aldehyde substrate in ethyl acetate, selecting appropriate internal standard for different chain length substrate. Standard was added to 45 μM, and finally reactions were extracted with 500 μL of methyl tert-butyl ether (MTBE). We choose different chain-length aldehyde (nonanal, dodecanal and tetradecanal) as substrate. Reaction products were also detected by GC-MS.

**Physiological Effects of Long-chain Aliphatic Compounds on Cyanobacteria**

Aliphatic compounds containing 12 carbons (dodecane, dodecanol, dodecanal, and dodecanoic acid) were chosen to investigate their effects on Syn6803. Firstly, 200 μL dodecane, dodecanol, and dodecanoic acid was added to the Syn6803 cultures, respectively. While 100 μL dodecanal was added to the Syn6803 cultures (System 1, **Supplementary Table 2**). Next, we further investigated the effect of high concentrations of alkanes on the growth of Syn6803.120 μM dodecanal, dodecanol and dodecanoic acid was added to the cultures of Syn6803, respectively. While 800 μM dodecane, which was 6.7 times to other aliphatic compounds, was added to the Syn6803 cultures (System 2, **Supplementary Table 2**). At 24 h, the whole cell absorption of 400 nm to 700 nm for differently treated Syn6803 was measured to analyze the effects of different aliphatic compounds on Syn6803.

**Supplementary Figures**


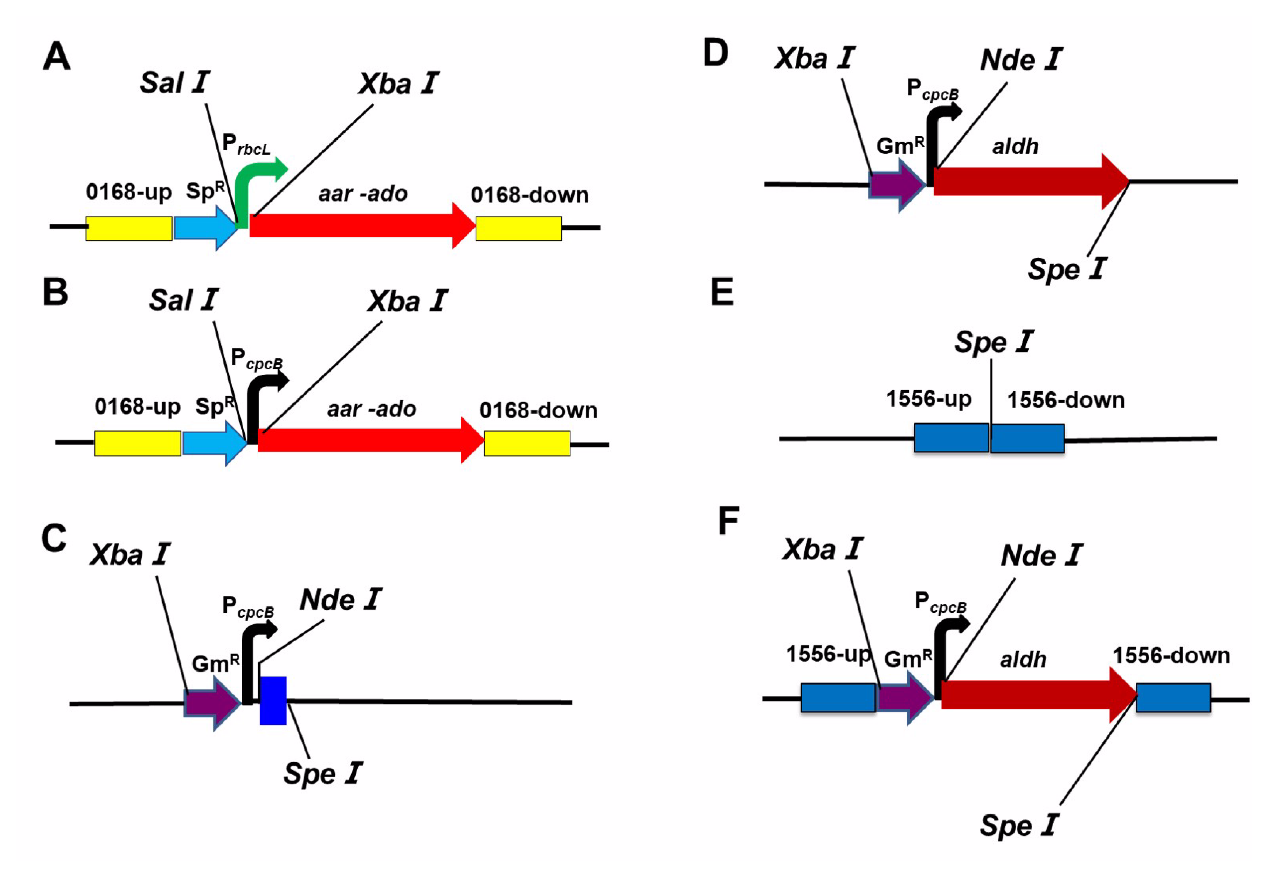


**Supplementary Figure 1 |** Schematic diagram of plasmids constructed in this study. **(A)** Plasmid pLX3. **(B)** The *aar-ado* overexpression plasmid pQY1 was constructed by replacing the promoter P*_rbcL_* with P*_cpcB_*. **(C, D, E)** Plasmid pZZ076, pQY6, and pZZ089 **(F)** The *aldh* overexpression plasmid pQY5 was constructed on the basis of the pZZ076, pQY6, and pZZ089.


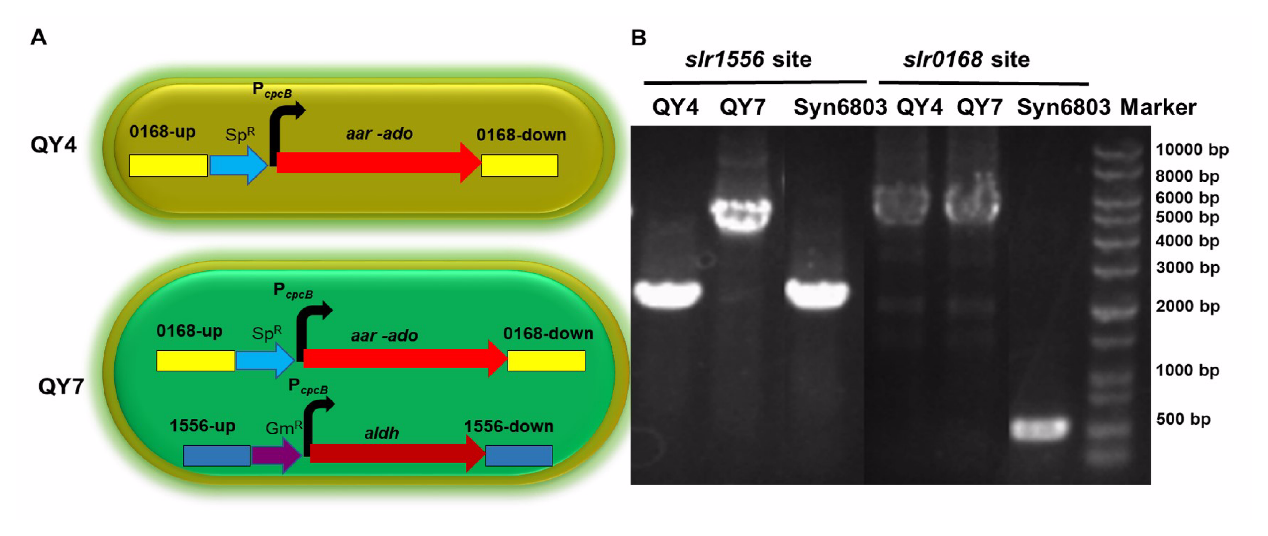


**Supplementary Figure 2 |** Schematic diagram and genotype verification of QY4 and QY7 strain. **(A)** Schematic diagram of Syn6803 mutants QY4 and QY7. **(B)** Genotype verification of QY4 and QY7 strain by PCR.


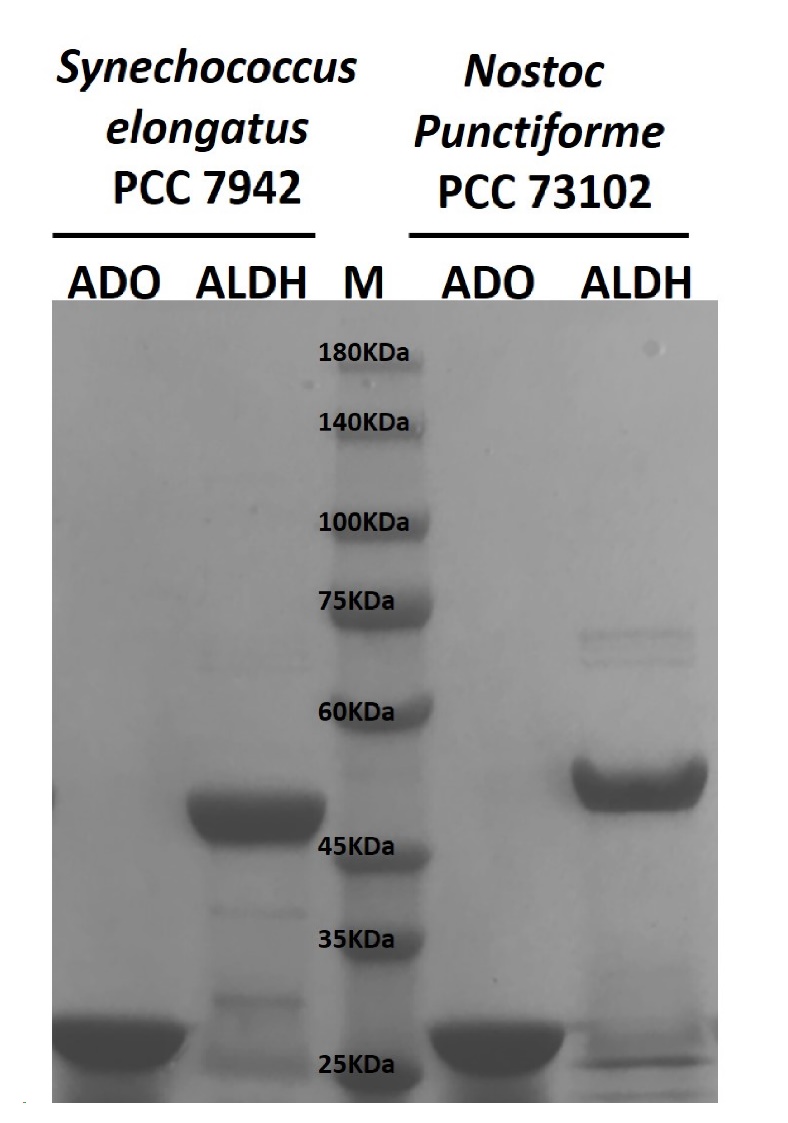


**Supplementary Figure 3 |** Purified ADO and ALDH from Syn7942 and Nos73102 were resolved in 12% SDS-PAGE. The recombinant ADO and ALDH from Syn7942 and Nos73102 were purified respectively. Recombinant proteins were purified as described in MATERIALS AND METHODS, and resolved in 12% SDS-PAGE with Coomassie Blue staining.


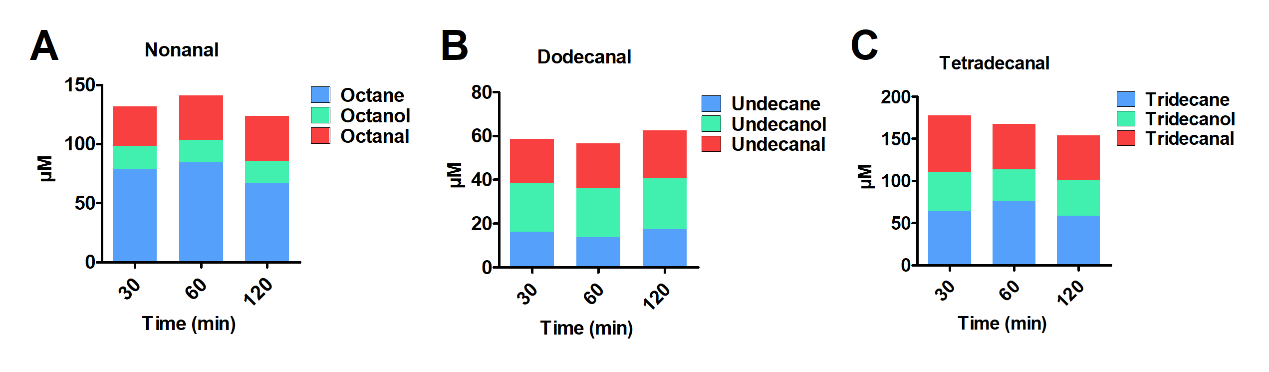


**Supplementary Figure 4 |** The ADO reactions with substrates of different carbon chain lengths. For further confirmation of the hydroxylase activity of ADO, according to the published procedure, enzyme assays were carried out in 500 μL reaction volumes in 1.5 mL GC vials shaken at 100 rpm at room temperature for 30 min. The reaction system included 30 μM ADO from Syn7942, 100 mM HEPES (pH7.4), 80 μM ferrous ammonium sulfate, 75 mM phenazine methosulfate (PMS) with 2 mM NADH, 500 μM aldehyde substrate in ethyl acetate. Appropriate internal standard was selected for different chain length substrates. Standard was added to 45 μM, and finally reactions were extracted with 500 μL of methyl tert-butyl ether (MTBE). Aldehydes of different chain lengths were chosen as the substrate: (**A**) nonanal, (**B**) dodecanal and (**C**) tetradecanal. Detection of the reaction products can be found in the MATERIALS AND METHODS.

**
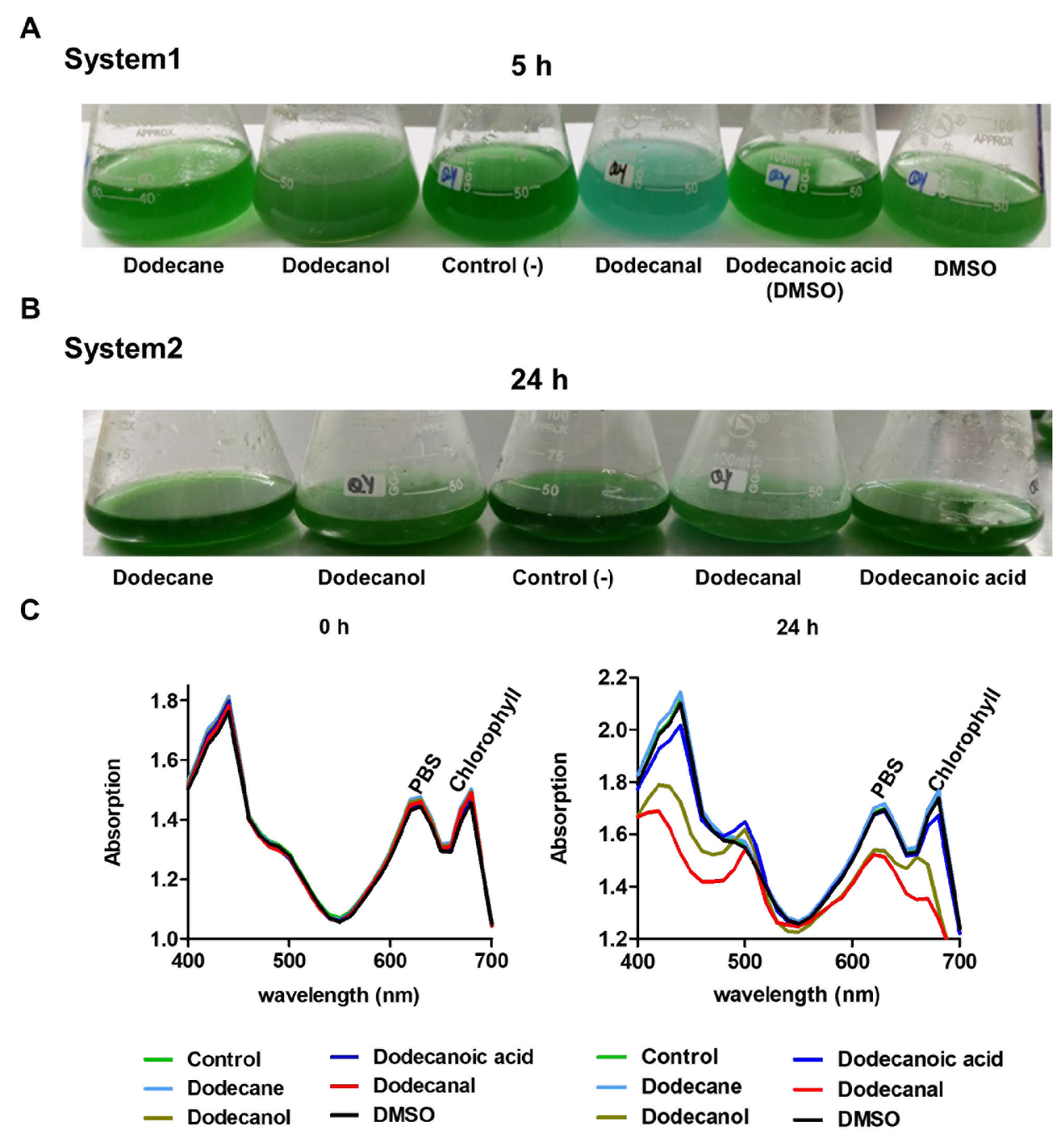
**

**Supplementary Figure 5 |** Effects of aliphatic compounds on Syn6803. **(A)** The phenotype of Syn6803 after the high concentrations of aliphatic compound ( System 1, **Supplementary Table 2**) was added for 5 h. **(B)** The phenotype of Syn6803 after the aliphatic compound (System 2, **Supplementary Table 2**) was added for 24 h. **(C)** Whole cell absorption spectra from 400 nm to 750 nm for Syn6803 when the C_12_ aliphatic compound was respectively added at 0 h and 24 h.

**Supplementary Tables**

**Supplementary Table** 1 **|** Oligonucleotide primers used in this work.

| **Primer** | **Sequence** |
| --- | --- |
| Synpcc7942_0489-Fsac1 | GAGCTCATGACTGCTGTCGTTCTCCC |
| Synpcc7942_0489-Rhind3 | AAGCTTCTAGAGCTTGCGGAAGAGGT |
| Npun_F0840-Fsac1 | GAGCTCATGATTACCACTGAATTATC |
| Npun_F0840-Rsal1 | GTCGACTTAACCCAAAAGTTTCTTTA |
| PcpcB-Fsal1 | GTCGACTTCGTTATAAAATAAACTTA |
| PcpcB-Rxba1 | TCTAGAATTAATCTCCTACTTGACTT |
| ddh-F | CAGAGGAGTTTGTCATAGGAGC |
| ddh-R | TACCCATTTCAACCTCAACGAT |
| slr0091-Fnde1 | CATATGATGAATACTGCTAAAACTG |
| slr0091-Rspe1 | ACTAGTCTAGGAGAACAACTTTTTG |
| 0168-1 | ACCTCTCCACGCTGAATTAG |
| 0168-2 | TTCCAGGCCACATTGTTGTC |

**Supplementary Table 2 |** The amounts of aliphatic compounds added to the Syn6803 cultures.

The amount of dodecanal is about half of the other three aliphatic compounds in System 1, and the amount of dodecane is 6.7 times of the other three aliphatic compounds in System 2.

|  | **System 1** | | **System 2** | |
| --- | --- | --- | --- | --- |
|  | volume (μL) | μmol | volume (μL) | μmol |
| dodecane | 200 | 880.59 | 182 | 800 |
| dodecanol | 200 | 894.11 | 27 | 120 |
| dodecanal | 100 | 450.31 | 27 | 120 |
| dodecanoic acid+DMSO | 800 | 828.67 | 96 | 120 |
| DMSO | 800 |  | 96 |  |
